# Differentially Expressed Transcripts and Dysregulated Signaling Pathways and Networks in African American Breast Cancer

**DOI:** 10.1371/journal.pone.0082460

**Published:** 2013-12-04

**Authors:** Paul A. Stewart, Jennifer Luks, Mark D. Roycik, Qing-Xiang Amy Sang, Jinfeng Zhang

**Affiliations:** 1 Department of Chemistry and Biochemistry, Florida State University, Tallahassee, Florida, United States of America; 2 Institute of Molecular Biophysics, Florida State University, Tallahassee, Florida, United States of America; 3 Department of Statistics, Florida State University, Tallahassee, Florida, United States of America; Stony Brook University, United States of America

## Abstract

African Americans (AAs) have higher mortality rate from breast cancer than that of Caucasian Americans (CAs) even when socioeconomic factors are accounted for. To better understand the driving biological factors of this health disparity, we performed a comprehensive differential gene expression analysis, including subtype- and stage-specific analysis, using the breast cancer data in the Cancer Genome Atlas (TCGA). In total, 674 unique genes and other transcripts were found differentially expressed between these two populations. The numbers of differentially expressed genes between AA and CA patients increased in each stage of tumor progression: there were 26 in stage I, 161 in stage II, and 223 in stage III. Resistin, a gene that is linked to obesity, insulin resistance, and breast cancer, was expressed more than four times higher in AA tumors. An uncharacterized, long, non-coding RNA, LOC90784, was down-regulated in AA tumors, and its expression was inversely related to cancer stage and was the lowest in triple negative AA breast tumors. Network analysis showed increased expression of a majority of components in p53 and BRCA1 subnetworks in AA breast tumor samples, and members of the aurora B and polo-like kinase signaling pathways were also highly expressed. Higher gene expression diversity was observed in more advanced stage breast tumors suggesting increased genomic instability during tumor progression. Amplified resistin expression may indicate insulin-resistant type II diabetes and obesity are associated with AA breast cancer. Expression of LOC90784 may have a protective effect on breast cancer patients, and its loss, particularly in triple negative breast cancer, could be having detrimental effects. This work helps elucidate molecular mechanisms of breast cancer health disparity and identifies putative biomarkers and therapeutic targets such as resistin, and the aurora B and polo-like kinase signaling pathways for treating AA breast cancer patients.

## Introduction

In 2013, more than 200,000 women in the United States will be diagnosed with breast cancer (BRCa) [1]. Despite advances in treatment and earlier detection, nearly 40,000 women with BRCa will die from this disease [1]. In clinical practice, BRCa is divided into subgroups based on the expression of the estrogen receptor (ER), progesterone receptor (PR), and the status of gene amplification of human epidermal growth factor 2 receptor (HER2). The four predominant subtypes reported extensively in literature are categorized based on these receptors: luminal A (ER+ and/or PR+, HER2-), luminal B (ER+ and/or PR+, HER2+), HER2 (ER-, PR-, HER2+), and triple negative (ER-, PR-, HER2-). The luminal A and luminal B subtypes express ER or PR and have several treatment options due to their susceptibility to hormone-based adjuvant therapies that target these receptors, but the HER2 subtype, as its name implies, only has HER2 amplification which severely limits adjuvant treatment options [2]. Triple negative breast cancer (TNBC) lacks expression of ER and PR, and HER2 amplification and treatment is primarily limited to surgery and chemotherapy [3]. A subtype of TNBC is the basal or basal-like phenotype that is negative for all three receptors but expresses specific basal markers (cytokeratin 5/6, cytokeratin 14, cytokeratin 17, and epidermal growth factor receptor) [4]. This phenotype is more likely to undergo metastasis and is associated with poorer prognosis [5]. 

African American (AA) women have a higher mortality than Caucasian American (CA) women with BRCa [1]. Although socioeconomic status has been shown to be an independent predictor of mortality, the increase in mortality of AA women with BRCa cannot be explained through socioeconomic status alone [6-8]. AA women still suffered from higher mortality in studies and in a randomized clinical trial where AA and CA women had equal access to care [9-11]. These observations suggest a biological basis for the elevated mortality of AA women, and this hypothesis is supported by a study that shows AA women are more likely to develop tumors with worse pathological characteristics (e.g., larger tumor size, less differentiation of cancerous cells) [12]. Other studies have shown that AA breast cancer patients are more likely to have detrimental tumor subtypes such as ER- and PR-, triple negative, and basal-like breast tumors [13-18].

There is no proven cause of health disparity, but studies indicate that genetic differences between AA and CA tumors do exist. Martin et al. first performed genome-wide mRNA expression analysis of tumor epithelium from 18 AA and 17 CA patients [19]. They found over 400 differentially expressed genes and identified a two-gene signature, putative phosphoserine phosphatase-like protein (PSPHL) and beta-crystallin B2 (CRYBB2), which could distinguish between populations. Field et al. conducted a study with 26 pairs of matched AA and CA BRCa patients and showed that molecular profiles differ between AA and CA breast tumors despite being matched by pathological characteristics. They detected more than two dozen differentially expressed genes that are involved in cellular growth, differentiation, invasion, metastasis, and immune response [20]. Additionally, a study by Grunda et al. using 12 pairs of matched AA and CA BRCa patients assessed a set of 84 genes involved in breast carcinoma prognosis, response to therapy, estrogen signaling, and tumor aggressiveness of age- and stage-matched AA and CA tumor samples [21]. They identified 20 differentially expressed genes from this set, leading to their conclusion that gene expression differences may play a role in increased metastatic potential, resistance to therapy, and worse clinical outcome in AA women.

These previous reports are consistent with one exception: ribosomal protein L13 (RPL13) was lower in AA women in the Martin et al. study but higher in the Field et al. study [19,20]. This could be explained by the small sample size or possibly by variation in age, stage, or subtype between the study populations. Moreover, these studies did not analyze differentially expressed genes by stage or subtype, possibly due to lack of patient samples. Since AA women are more likely to be afflicted by worse subtypes it is apparent that comprehensive studies that include stage and subtype analyses as well as larger sample size are necessary to elucidate the genetic factors responsible for BRCa health disparity.

We used next generation sequencing (NGS) data from the Cancer Genome Atlas (TCGA) to determine differentially expressed genes and other transcripts between a large number of age- and stage-matched AA and CA patient primary tumor samples. We chose NGS data because of its accuracy and the ability to use it to determine differential expression between non-coding RNAs in addition to coding RNAs. This study represents, to our knowledge, the first time that NGS data was used in combination with a large patient cohort to investigate expression differences of genes and other transcripts between AA and CA BRCa patients, and to our knowledge, this work represents the largest and most comprehensive study on health disparity in AA BRCa to date. To our knowledge, we are the first to identify differentially expressed genes by stage and subtype between AA and CA tumors in addition to differentially expressed subnetworks and pathways. 

There are several options to test for differential expression. The Poisson distribution and the negative binomial distribution are both discrete probability distributions that can model count data from a gene sequencer over a finite period of time, and many previous methods for differential expression analysis of NGS data have been based on the Poisson distribution [22,23]. However, the Poisson distribution often under-estimates the actual variations of expression data because it assumes the mean is equal to the variance [24,25]. The negative binomial distribution can tolerate instances of large biological variance between replicates and is often used to cope with the over-dispersion issue by allowing mean and variance to be different. With this in mind, we chose to use DESeq, an R Bioconductor package, for our analyses because it uses the negative binomial distribution to derive a test for differential expression [26,27].

## Results

Table **1** shows the breakdown of patient demographics obtained from TCGA. AA women had nearly double the mortality of their age- and stage-matched CA counterparts with 18.87% mortality compared to 10.28% CA mortality, and this increase in mortality is despite patient samples being age- and stage-matched. As mentioned in the Introduction, this disparity is likely caused by both socioeconomic and biological factors. Information regarding socioeconomic status or access to care is not available at TCGA. Our observed mortalities are lower than previously reported population data for both races: 18.2% for CA women and 26.8% for AA women [1]. This is likely caused by comparably fewer patients with late stage breast cancer donating their tumor samples to TCGA. 

**Table 1:** Breast cancer patient clinical data from TCGA.

**Characteristics**	**Caucasian American (n=574)**		**African American (n=53)**		**Fisher's Exact Test**
	**Number**	**Pct.**	**Number**	**Pct.**	**P-value**
**Age**					0.603
**< 50**	157	27.35%	18	33.96%	
**50-64**	252	43.90%	21	39.62%	
**65+**	165	28.75%	14	26.42%	
**Tumor Stage**					0.783
**1**	117	20.38%	11	20.75%	
**2**	337	58.71%	29	54.72%	
**3**	120	20.91%	13	24.53%	
**Tumor Type**					0.003
**Luminal A**	243	42.33%	16	30.19%	
**Luminal B**	62	10.80%	0	0.00%	
**Triple Negative**	67	11.67%	10	18.87%	
**HER2 Type**	17	2.96%	3	5.66%	
**Other/NA**	185	32.23%	24	45.28%	
**Vital Status**					0.066
**Living**	515	89.72%	43	81.13%	
**Deceased**	59	10.28%	10	18.87%	

**Figure 1:**
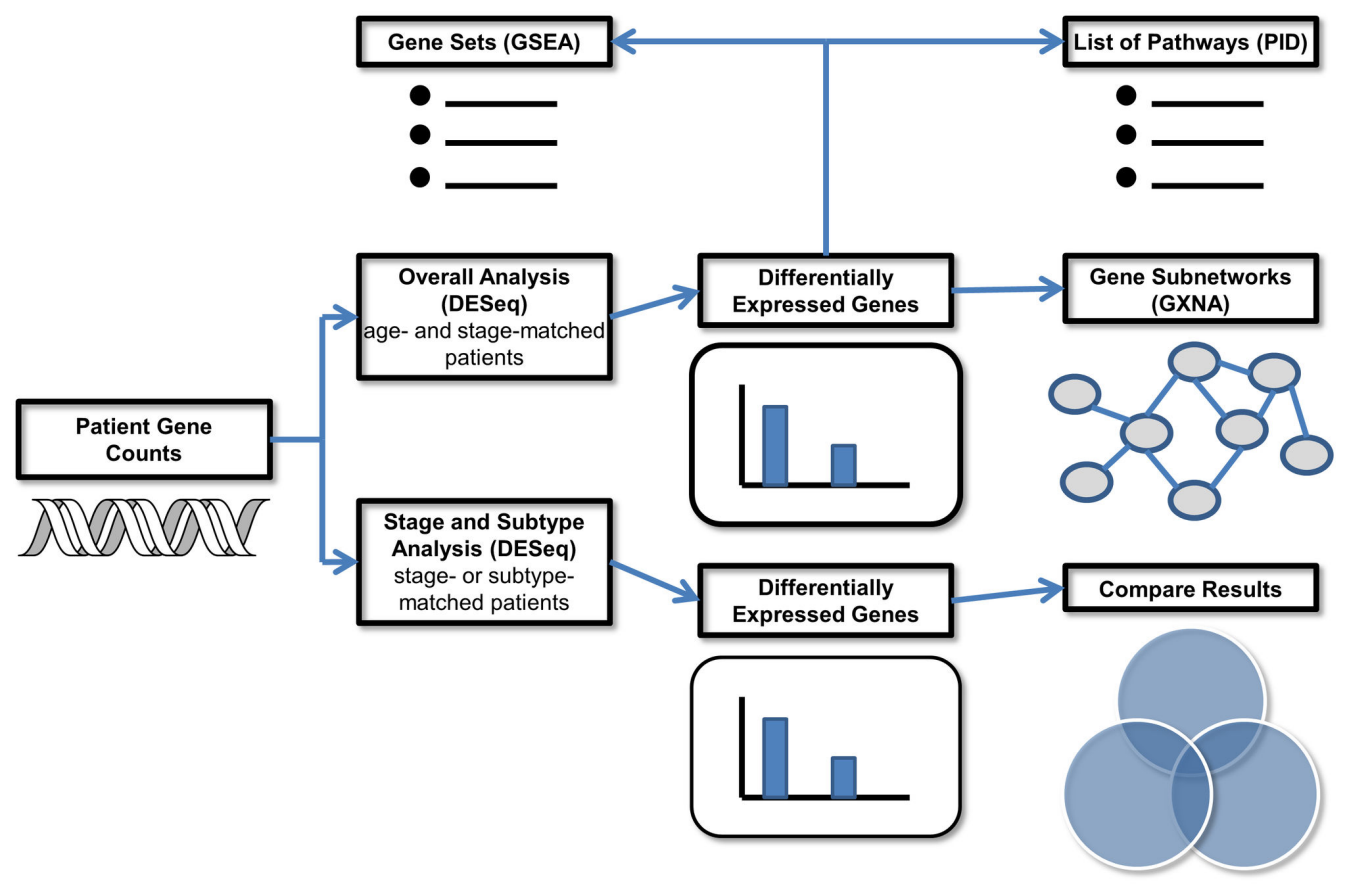
A flowchart summarizing the data analysis methodology.

Differential expression analysis at the individual gene level was firstly conducted to identify genes and other transcripts that differ between AA and CA BRCa patients using DESeq; secondly, pathway analysis and gene set enrichment analysis with the Pathway Interaction Database (PID) and Gene Set Enrichment Analysis (GSEA) was performed; thirdly, Gene Expression Network Analysis (GXNA) was used to identify differentially expressed subnetworks; finally, patients were stratified to study differences in stage- or subtype-specific gene expression again with DESeq (Figure **1**).

In total, 674 unique genes and other transcripts were shown to be differentially expressed between the various comparisons performed (Table **S1**). The number of unique genes was computed by combining gene names from the overall, stages I-III, luminal A, HER2, and triple negative comparisons into a single list. We then removed duplicate gene names from the list, and this new list without duplicates was then counted to give the total number of unique genes. To identify trends in gene expression, primary breast tumor expression data between AA and CA patients irrespective of subtype or stage (i.e., the “overall” comparison) was first compared. Three hundred forty-two genes and other transcripts, 1.7% of the 20,531 genes and other transcripts assessed, were identified by DESeq as differentially expressed between AA and CA primary breast tumors (P < 0.001, Table **S2**). One hundred ten of these genes and other transcripts showed increased expression in AA tumors while 232 were decreased. Thirty-seven of the 342 genes and other transcripts exhibited significant differential expression (i.e., log_2_ fold-change greater than 1.0 or less than -1.0), and selected genes and other transcripts with high fold-change and relevance to breast cancer can be seen in Table **2**. 

**Table 2:** DESeq results showing significant changes of gene expression in age- and stage-matched AA tumors.

**Gene**	**Log_2_ Fold-Change**	**P-value**	**P-adj**	**Relevance**	**Citation**
RETN	2.25	3.05E-06	2.80E-03	Expressed in inflammatory cells; insulin resistance in mice; increased in BRCa patients; high expression in breast cancer tissue associated with poor survival and malignant characteristics	[42-44]
**CRYBB2**	1.81	1.24E-13	1.25E-09	Differentially expressed in several studies investigating cancer health disparity	[19,20,37,38]
TREML4	1.79	8.87E-08	1.83E-04	Elicits T-cell immunity and tumor protection when coupled with the breast cancer antigen HER2	[72]
**CXCL10**	1.48	1.67E-04	2.87E-02	Chemokine; involved in breast tumor invasiveness and progression	[73]
PAX6	1.33	1.89E-05	9.57E-03	Facilitates regulatory roles in breast cancer cell line proliferation and tumor progression	[74]
BMP6	1.25	2.63E-04	3.59E-02	Promotes E-cadherin expression through repressing delta-EF1 in breast cancer cells; abnormally expressed and regulated by estrogen receptor alpha in breast cancer cells	[75,76]
EMR1	1.19	2.51E-04	3.48E-02	Hormone receptor; overexpressed in a cluster of genes associated with poor prognosis in breast cancer	[77]
APOBEC3B	1.13	1.10E-04	2.43E-02	Enzymatic source of mutation in breast cancer	[78]
SPATA18	-1.28	7.66E-06	5.53E-03	p53-inducible protein; controls mitochondrial quality	[79]
ADAMTS15	-1.29	1.82E-04	2.95E-02	Metalloprotease known to inhibit breast cancer cell migration	[49]
ABCC6	-1.37	1.10E-04	2.43E-02	Multidrug resistance-associated protein	[80]

Log_2_ fold-change is for mean AA expression relative to mean CA expression. Log_2_-fold change is calculated as log_2_(AA/CA). Bold entries have been observed as differentially expressed previously in literature. Gene names: ABCC6 - ATP binding cassette, sub family C (CFTR/MRP), member 6; ADAMTS15 - ADAM metallopeptidase with thrombospondin type 1 motif, 15; APOBEC3B - apolipoprotein B mRNA editing enzyme, catalytic polypeptide like 3B; BMP6 - bone morphogenetic protein 6; CRYBB2 - crystallin, beta B2; CXCL10 - chemokine (C X C motif) ligand 10; EMR1 - egf like module containing, mucin like, hormone receptor like 1; PAX6 - paired box 6;RETN - resistin; SPATA18 - spermatogenesis associated 18; TREML4 - triggering receptor expressed on myeloid cells like 4.

The 342 differentially expressed genes and other transcripts from the overall comparison were searched against PID (Table **3**). PID places genes into known pathways and ranks them by the probability that their associated genes are enriched in the input gene list, and a low P-value indicates a greater chance of enrichment. The top PID pathway was the tumor protein p73 (p73) transcription factor network (P = 4.75E-05). p73 is a tumor suppressor whose overexpression is seen in some aggressive breast cancer tumors and breast cancer cell lines [28]. The next most significant PID result was the aurora B signaling pathway (P = 1.07E-04). Aurora kinase B (AURKB) is a mitotic checkpoint kinase that mediates chromosome segregation and is overexpressed in a variety of cancers including lung, colorectal, and prostate [29-31]. The polo-like kinase (PLK1) signaling events pathway was also identified by PID (P = 2.35E-04). PLK1 is a kinase significantly overexpressed in TNBC that may be a potential therapeutic target for TNBC [32]. We saw differential expression in the BRCA1 associated RING domain 1 (BARD1) signaling events pathway (P = 4.70E-04). BARD1 interacts with tumor protein p53 (p53 or TP53); breast cancer 1, early onset (BRCA1); and breast cancer 2, early onset (BRCA2), all of which are important tumor suppressors in breast cancer [33-35]. The genes within the pathways identified by PID had almost unanimous increase in gene expression in AA tumors. The one exception was BARD1 signaling events pathway which had one gene, RAD50 homolog (S. cerevisiae) (RAD50), decrease in AA tumors. Additionally, we uploaded the genes from the Martin et al., Field et al., and Grunda et al. studies on health disparity to PID and compared the results with our own [19-21]. Out of all the previous works, our results only had the aurora B signaling pathway in common with the Martin et al. data [19].

**Table 3:** Differentially expressed genes in AA tumors are part of pathways with relevance to breast cancer.

**Pathway Name**	**Genes and Transcripts**	**P-value**	**P-adj**
p73 transcription factor network	ADA, AEN, BUB1, CDK1, HSF1, PML, RAD51	4.75E-05	1.62E-02
Aurora B signaling	AURKB, BUB1, INCENP, KIF20A, KIF23	1.07E-04	1.83E-02
PLK1 signaling events	BUB1, CDC25B, CDK1, INCENP, KIF20A	2.35E-04	2.68E-02
Aurora C signaling	AURKB, INCENP	4.68E-04	3.21E-02
BARD1 signaling events	EWSR1, FANCA, RAD50, RAD51	4.70E-04	3.21E-02

The underlined entry was the only gene not increased in AA samples. Results from the Pathway Interaction Database (http://pid.nci.nih.gov/). Gene names: ADA - adenosine deaminase; AEN - apoptosis enhancing nuclease; AURKB - aurora kinase B; BUB1 - BUB1 mitotic checkpoint serine/threonine kinase; CDC25B - cell division cycle 25B; CDK1 - cyclin-dependent kinase 1; EWSR1 - EWS RNA-binding protein 1; FANCA - Fanconi anemia, complementation group A; HSF1 - heat shock transcription factor 1; INCENP - inner centromere protein antigens 135/155kDa; KIF20A - kinesin family member 20A; KIF23 - kinesin family member 23; PML - promyelocytic leukemia; RAD50 - RAD50 homolog (S. cerevisiae); RAD51 - RAD51 recombinase.

**Figure 2:**
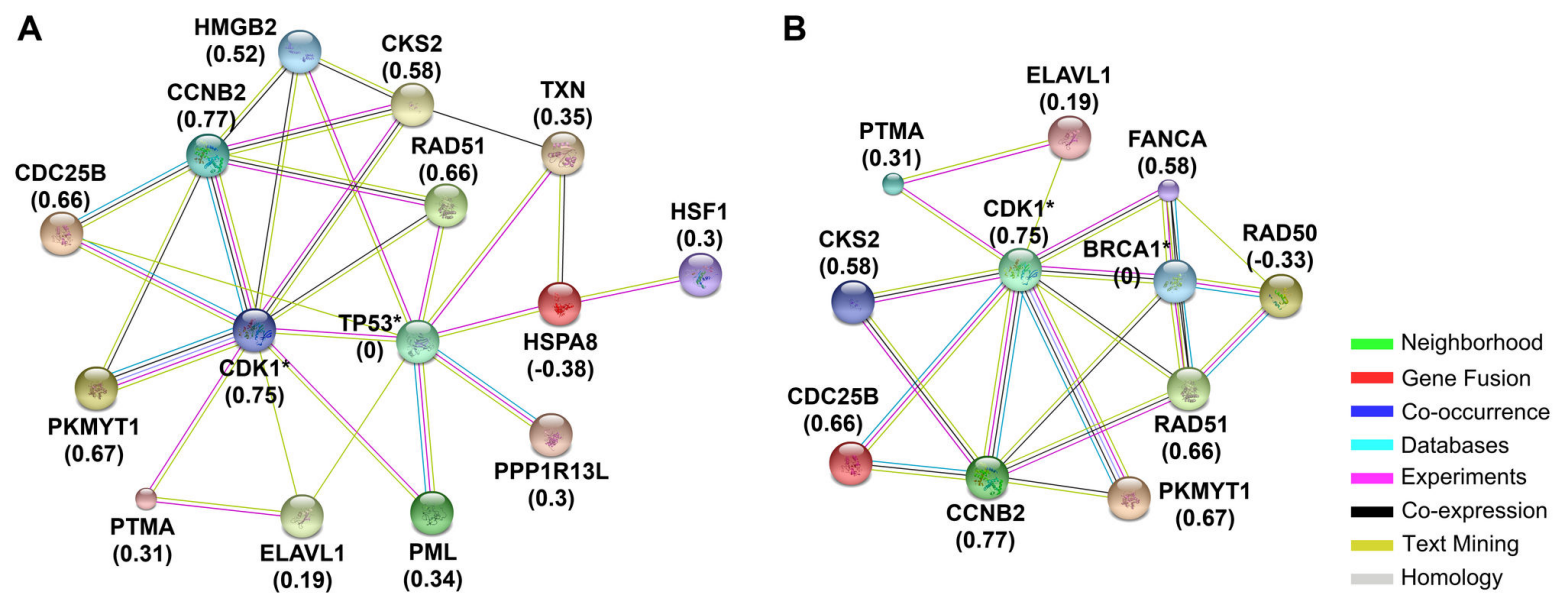
Differentially expressed subnetworks identified by Gene Expression Network Analysis. Subnetworks containing p53 (A) and BRCA1 (B) were differentially expressed in AA tumors. Subnetworks were identified using GXNA and visualized using STRING. Starred results were not differentially expressed but were included in the subnetwork by GXNA. Values in parentheses are the mean fold changes of log_2_-transformed AA expression relative to CA expression, calculated as log_2_(CA)/log_2_(AA). Gene names: HMGB2: BRCA1 - breast cancer 1, early onset; CCNB2 - cyclin B2; CDC25B - cell division cycle 25B; CDK1 - cyclin-dependent kinase 1; CKS2 - CDC28 protein kinase regulatory subunit 2; ELAVL1 - ELAV (embryonic lethal, abnormal vision, Drosophila)-like 1 (Hu antigen R); FANCA - Fanconi anemia, complementation group A; HMGB2 - high mobility group box 2; HSF1 - heat shock transcription factor 1; HSPA8 - heat shock 70kDa protein 8; PKMYT1 - protein kinase, membrane. associated tyrosine/threonine 1; PML - promyelocytic leukemia; PPP1R13L - protein phosphatase 1, regulatory subunit 13 like; PTMA - prothymosin, alpha; RAD50 - RAD50 homolog (S. cerevisiae); RAD51 - RAD51 recombinase; TP53 - tumor protein p53; TXN – thioredoxin.

GXNA was then used to identify differentially expressed subnetworks between AA and CA tumors. GXNA takes patient expression data as input and then outputs a ranked list of subnetworks that contain interactions involving the differentially expressed genes. GXNA identified two subnetworks, one involving p53 and another involving BRCA1, both with a majority of components upregulated in AA breast tumors (P < 0.001, Figure **2**). STRING was used to visualize GXNA data and to show potential gene product interactions beyond what is shown in the GXNA interactions. Gene Set Enrichment Analysis did not identify any differentially expressed gene sets (P-value > 0.05 or with unacceptable FDR; results not shown).

**Figure 3:**
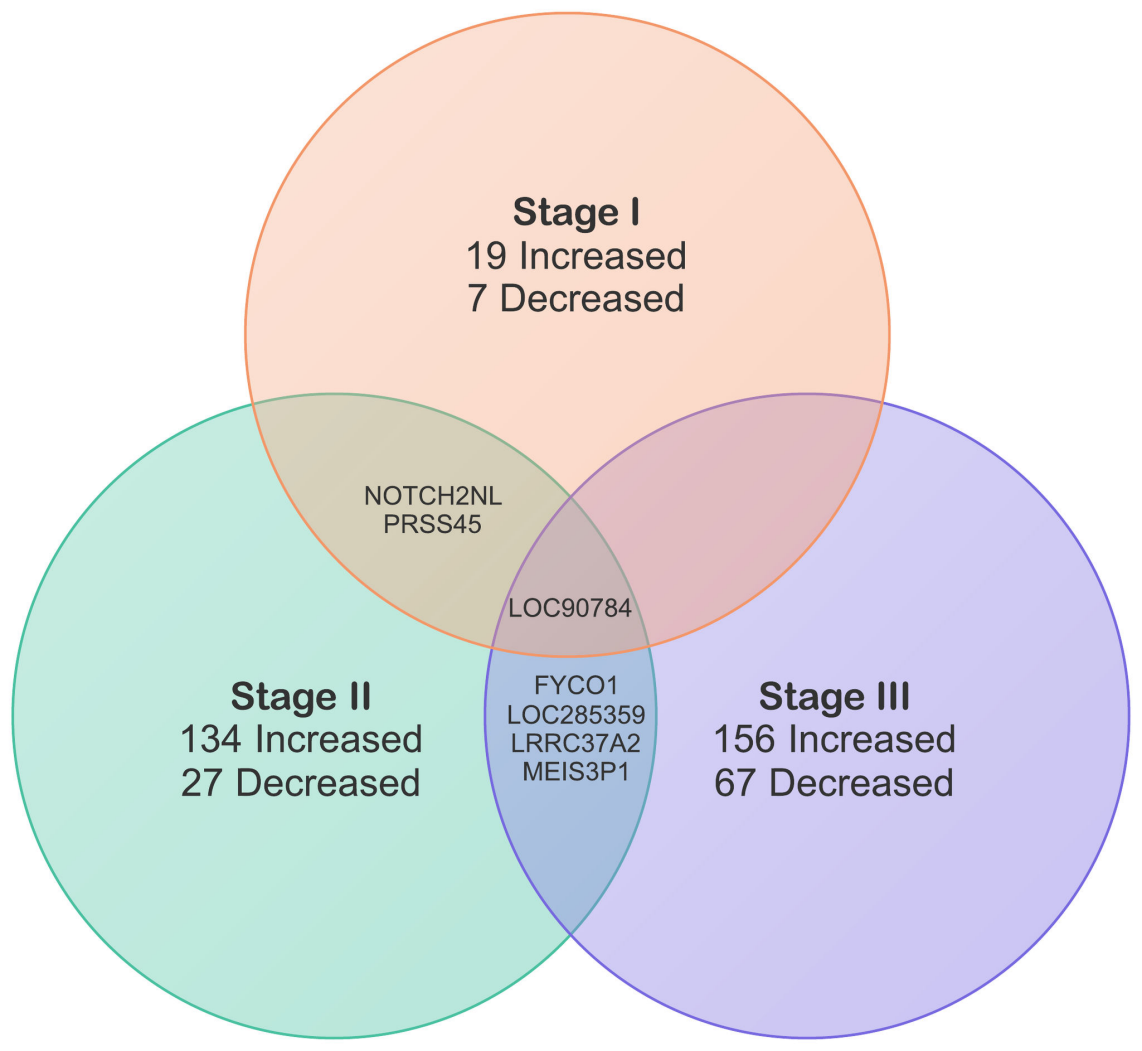
Venn diagram depicting overlap of differentially expressed genes and other transcripts between stage-matched African- and Caucasian-American tumors. Increases or decreases indicate AA gene expression and are relative to CA expression. Gene names: FYCO1 - FYVE and coiled-coil domain containing 1; LOC90784 - Not Available; LOC285359 - Not Available; LRRC37A2 - leucine rich repeat containing 37, member A2; MEIS3P1 - Meis homeobox 3 pseudogene 1; NOTCH2NL - notch 2 N-terminal like; PRSS45 - protease, serine, 45.

Stage-matched and then subtype-matched AA and CA tumors were next compared, but a lack of stage IV and luminal B AA tumors allowed us to only evaluate stages I through III and the luminal A, HER2, and triple-negative subtypes. Selected results from these comparisons are summarized in Table **4**. Interestingly, very few genes and other transcripts overlapped between the stage comparisons (Figure **3**). Twenty-six genes and other transcripts were differentially expressed in the stage I comparison (19 increased and 7 decreased in AA tumors, Table **S3A**), including BUB1 mitotic checkpoint serine/threonine kinase (BUB1; 1.05 log_2_ fold-change; P = 9.80E-04) which is a potent prognostic factor for human breast cancer [36]. One hundred sixty-one genes were differentially expressed in the stage II comparison (134 increased and 27 decreased, Table **S3B**) including crystallin, beta B2 (CRYBB2; 2.00 log_2_ fold-change; P = 3.27E-10) which was differentially expressed in AA breast, colorectal, and prostate cancers [19,20,37,38]. Two hundred twenty-three genes were differentially expressed in the stage III comparison (156 increased and 67 decreased, Table **S3C**), including estrogen receptor 1 (ESR1; -2.25 log_2_ fold-change; P = 8.80E-05). Forty-six genes and other transcripts were differentially expressed in the luminal A comparison (39 increased and 7 decreased in AA tumors, Table **S3D**) including polycystin (PKD) family receptor for egg jelly (PKDREJ; -1.18 log_2_ fold-change; P = 9.56E-04) which is point mutated in breast cancer [39]. Twenty-five genes and other transcripts were differentially expressed in the HER2 comparison (21 increased and 4 decreased, Table **S3E**) including TCRgamma alternative reading frame protein (TARP; 8.74 log_2_ fold-change; P = 2.31E-04) which is a breast and prostate tumor-associated antigen [40]. Fifteen genes and other transcripts were differentially expressed in the triple negative comparison (10 genes increased and 5 decreased, Table **S3F**) including peptidoglycan recognition protein 1 (PGLYRP1; 2.99 log_2_ fold-change; P = 7.64E-04) whose expression may reflect immune cell response to developing breast tumors [41].

**Table 4:** DESeq results showing significant changes of gene expression in subtype- or stage-matched AA tumors.

**Gene**	**Stage/Subtype**	**Log_2_ Fold-Change**	**P-value**	**P-adj**	**Relevance**	**Citation**
TARP	HER2	8.74	2.31E-04	5.01E-01	Breast and prostate tumor-associated antigen	[40]
PKDREJ	Luminal A	-1.18	9.56E-04	4.20E-01	Point mutated in breast cancers	[39]
PGLYRP1	Triple negative	2.99	7.64E-04	1.00E+00	Increased in expression may reflect immune cell response to developing breast tumor	[41]
**BUB1**	Stage I	1.05	9.80E-04	7.44E-01	Nuclear localization is a potent prognostic factor for human breast cancer	[36]
GALNT6	Stage I	-1.45	3.00E-04	4.03E-01	Deregulation of GALNT6 gene could be an early event during human breast carcinogenesis	[81]
**CRYBB2**	Stage II	2.00	3.27E-10	6.60E-06	Differentially expressed in several studies investigating cancer health disparity	[19,20,37,38]
FLJ45983	Stage III	-2.40	2.21E-04	5.66E-02	Putative uncharacterized protein; Hypermethylated in primary and metastatic tumors	[82]
**ESR1**	Stage III	-2.25	8.80E-05	4.91E-02	Estrogen receptor alpha	
FOXA1	Stage III	-1.15	3.48E-05	3.97E-02	Expression correlates with luminal subtype A breast cancer; significant predictor of cancer-specific survival in patients with ER-positive tumors	[83]
LRIG1	Stage III	-1.10	3.39E-04	6.11E-02	Estrogen-regulated growth suppressor; correlates with longer relapse-free survival in ERα-positive breast cancer; tumor suppressor	[84,85]
PLCG2	Stage III	1.00	1.08E-04	4.91E-02	Hypermethylated in metastatic breast cancer cell line	[86]

Log_2_ fold-change is for mean AA expression relative to mean CA expression. Log_2_-fold change is calculated as log_2_(AA/CA). Bold entries have been previously observed as differentially expressed in literature. Gene names: CRYBB2 - crystallin, beta B2; CYP4F8 - cytochrome P450, family 4, subfamily F, polypeptide 8; ESR1 - estrogen receptor 1; FLJ45983 - Not Available; FOXA1 - forkhead box A1; GALNT6 - UDP-N-acetyl-alpha-D-galactosamine:polypeptide N-acetylgalactosaminyltransferase 6 (GalNAc-T6); LRIG1 - leucine-rich repeats and immunoglobulin-like domains 1; PGLYRP1 - peptidoglycan recognition protein 1; PKDREJ - polycystin (PKD) family receptor for egg jelly; PLCG2 - phospholipase C, gamma 2 (phosphatidylinositol-specific), TARP - TCRgamma alternative reading frame protein.

**Figure 4:**
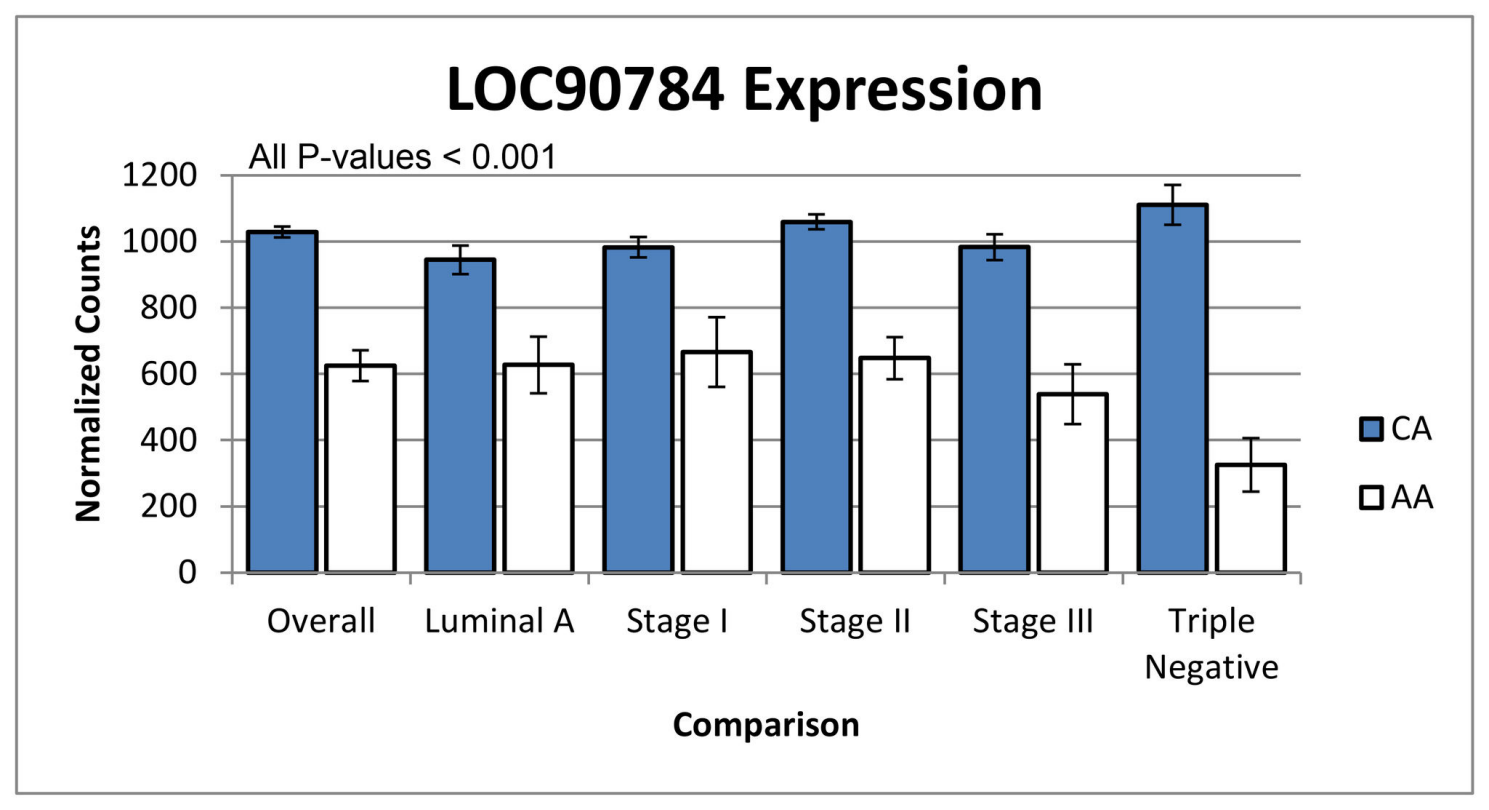
LOC90784, a long non-coding RNA, was differentially expressed across comparisons of African- and Caucasian-American tumors. The expression of this transcript was consistent in CA tumors, but its expression was inversely related to stage and was lowest in AA with TNBC. P-values for the comparisons were overall: 1.46E-14, luminal A: 1.98E-04, stage I: 6.61E-04, stage II: 3.63E-08, stage III: 2.57E-05, and triple negative: 1.86E-09 (see Supplementary Tables). Error bars are standard error.

LOC90784, a transcript, was the only gene or transcript differentially expressed in more than one subtype (luminal A and triple negative). Additionally, it was differentially expressed in the overall, stage I, stage II, and stage III comparisons. This made it the only transcript or gene we found to be differentially expressed across six different comparisons. Interestingly, the expression of LOC90784 remained consistent across CA tumor stage and grade but was consistently lower in AA samples, and LOC90784 expression was inversely related to stage among AA patient samples and was the lowest in triple negative AA tumors (Figure **4**).

These results share some agreement with previous studies (Table **5**). Fifteen genes out of 424 were found to be in common with those reported by Martin et al. [19]. An additional two genes were in common with those identified by Martin et al. but the results are contradictory (deoxyuridine triphosphatase or DUT expression was higher in AA samples in our results but lower in the Martin et al. results; Zic family member 1 or ZIC1 was lower in AA samples in our results but higher in the Martin et al. results) [19]. Three out of 27 genes were found to be in common with those found by Field et al. (CRYBB2, mitochondrial ribosomal protein L48 or MRPL48, and zinc finger protein 395 or ZNF395) [20], and one gene out of twenty was shared with those found by Grunda et al. (ESR1) [21]. It is not straightforward to assess the statistical significance of the agreements between these studies and our own so we cannot rule out that the agreements of our studies are purely by chance. 

**Table 5:** Comparison of increases and decreases in AA gene expression between presented results and previous findings.

**Gene**	**Results (674 Genes)**	**Martin (17 of 424 Genes)**	**Field (3 of 27 Genes)**	**Grunda (1 of 20 Genes)**
AK2	increase	increase		
APP	increase	increase		
BUB1	increase	increase		
CKS2	increase	increase		
CRYBB2	increase	increase	increase	
CXCL10	increase	increase		
DNAJC15	increase	increase		
*DUT*	increase	decrease		
ESPL1	increase	increase		
ESR1	decrease			decrease
HLA-DQB1	increase	increase		
ISG20	increase	increase		
KIF20A	increase	increase		
MRPL48	increase		increase	
SNED1	decrease	decrease		
TAP2	increase	increase		
UBE2C	increase	increase		
*ZIC1*	decrease	increase		
ZNF395	decrease	decrease	decrease	

Italicized genes were inconsistent with literature while blank entries were not observed. Comparisons are an increase in decrease in expression of AA samples relative to CA samples. The number of genes in parentheses refers to the number of genes in common with the presented results compared to the total number of differentially expressed genes from each study. Gene names: AK2 - adenylate kinase 2; APP - amyloid beta (A4) precursor protein; BUB1 - BUB1 mitotic checkpoint serine/threonine kinase; CKS2 - CDC28 protein kinase regulatory subunit 2; CRYBB2 - crystallin, beta B2; CXCL10 - chemokine (C-X-C motif) ligand 10; DNAJC15 - DnaJ (Hsp40) homolog, subfamily C, member 15; DUT - deoxyuridine triphosphatase; ESPL1 - extra spindle pole bodies homolog 1 (S. cerevisiae); ESR1 - estrogen receptor 1; HLA-DQB1 - major histocompatibility complex, class II, DQ beta 1; ISG20 - interferon stimulated exonuclease gene 20kDa; KIF20A - kinesin family member 20A; MRPL48 - mitochondrial ribosomal protein L48; SNED1 - sushi, nidogen and EGF-like domains 1; TAP2 - transporter 2, ATP-binding cassette, sub-family B (MDR/TAP); UBE2C - ubiquitin-conjugating enzyme E2C; ZIC1 - Zic family member 1; ZNF395 - zinc finger protein 395.

## Discussion

We identified 342 differentially expressed genes and other transcripts (P < 0.001) between AA and CA primary breast cancer tumors in the overall comparison. Thirty-seven of these exhibited significant change (i.e., log_2_ fold-change > 1.0 or < -1.0), and selected breast cancer-associated genes are shown (Table **2**). Several genes have increased expression in AA tumors, including a few with direct ties to breast cancer such as resistin (RETN). RETN is an adipocytokine whose expression level is significantly increased in AA tumors (2.25 log_2_ fold-change; P = 3.05E-06). It is expressed in inflammatory cells in humans and plays a role in insulin resistance in mice [42,43]. Breast cancer patients show increased resistin concentrations over control [44], and a recent study indicates that high resistin expression in breast cancer tissue is associated with decreased survival and more malignant characteristics [45]. Its presence at such high expression levels suggests that resistin may play an important role in AA BRCa because of the strong link between obesity and cancer mortality [46-48]. Likewise, a number of genes have reduced expression in AA women, including ADAM metallopeptidase with thrombospondin type 1 motif, 15 (ADAMTS15; -1.29 log_2_ fold-change; P = 1.82E-04). ADAMTS15 is a metalloprotease known to inhibit breast cancer cell migration [49], and it is possible that the reduced expression of this gene could have consequences in AA BRCa progression.

We noticed an increase in expression of CRYBB2 in AA samples (Table **2**). This gene encodes a protein that is a major component of the vertebrate eye lens and whose mutation may cause cataracts [50]. Expression of this gene was increased in AA tumor samples [19], and its expression was increased in both nonmalignant and tumor samples [20]. Martin et al. used this gene to differentiate between AA and CA patients while Field et al. hypothesized that overexpression of this gene could possibly enhance AA tumorigenesis. Expression of CRYBB2 was also increased in a study comparing AA and CA colorectal cancer and in another study comparing AA and CA prostate cancer [37,38]. These last two results are intriguing since health disparity exists in both prostate cancer and colorectal cancer in the AA population. The fact that this gene is overexpressed in nonmalignant tissues could simply be a result of racial differences, but we only observed differential expression of CRYBB2 in our overall and stage II comparisons. We would expect to see CRYBB2 in more comparisons if this gene was always differentially expressed between races. We suggest further studies into the role, if any, which CRYBB2 plays in cancer and health disparity.

We saw elevated expression of all members of the aurora B signaling pathway identified by PID (P = 1.07E-04). Aurora B kinase (AURKB), a member of the pathway sharing its name, had nearly doubled expression in AA compared to CA (0.89 log_2_ fold-change; P = 3.49E-05; Table **S2**). AURKB is a mitotic checkpoint kinase that mediates chromosome segregation and is overexpressed in a variety of cancers including lung, colorectal, and prostate [29-31], and it interacts with the aforementioned BARD1 pathway [35]. There has been no evidence that AURKB is overexpressed in breast cancer, but aurora kinase family inhibitors have been suggested as treatments for cancer. Breast cancer cell lines treated with the highly selective AURKB inhibitor Barasertib show anti-neoplastic effects [51-53]. Based on the increased expression of AURKB and other aurora B signaling pathway members in AA tumors, we speculate that this drug may be used as an effective treatment for African American breast cancer patients. 

We used GXNA to identify differentially expressed subnetworks in AA BRCa. Subnetworks differ from pathway analysis results because they consist of genes that share interactions with one-another across multiple pathways, and this allows for the identification of differentially expressed subnetworks that are not limited to a single pathway. We identified two differentially expressed subnetworks related to BRCa with GXNA, one centered on p53 (P = 0.001) while the other involved BRCA1 (P = 0.001, Figure **2**). Both of these pathways had a majority of genes with increased expression in AA samples. p53 is well-studied tumor suppressor that can activate DNA repair as well as initiate apoptosis [54], and p53 status is also an independent predictor of survival in AA women with BRCa [55]. Although p53 was not differentially expressed in our results, many of its signaling neighbors were increased. BRCA1 is an important DNA repair enzyme whose loss is associated with dramatically increased BRCa incidence [56], and BRCA1 and BRCA2 mutations are seen in high-risk AA women with BRCa [57]. Like p53, BRCA1 was not differentially expressed in our results but many of its signaling neighbors were increased. The GXNA results are strengthened by our pathway analysis result that showed increased activity in the BARD1 pathway whose members interact with both p53 and BRCA1 [33-35]. These results strongly suggest increased activity in the p53 and BRCA1 subnetworks in AA women, but these results are somewhat contradictory: higher expression or activity in two pathways related to tumor suppression and DNA repair should logically have a beneficial effect and should not contribute to health disparity. However, we suggest that there may be dysregulation up or downstream of these pathways as a result of a mutation, and the increase in activity of these pathways could be explained by the patient’s cells attempting to compensate for dysregulation. p53-expressing tumors are more frequent in AA women with TNBC when compared to their CA counterparts with TNBC [58], so the increase in expression of the p53 subnetwork could be partially explained by this fact.

Our stage III comparison showed that AA tumors had significantly lower ESR1 (-2.25 log_2_ fold-change; P = 8.80E-05; Table **4**). This comparison was our only comparison where ESR1 was differentially expressed between AA and CA samples, and Grunda et al. saw a similar decrease in expression of ESR1 in their study [21]. We believe this finding suggests that AA women may have reduced ER expression in later stages or that AA women may be more susceptible to mutations causing the ER- phenotype.

LOC90784, a transcript, was the only gene or transcript differentially expressed in the overall, stage I, stage II, stage III, luminal A, and triple negative comparisons. The expression of LOC90784 was very similar across the CA population regardless of stage or subtype, but its expression was inversely related to stage among AA samples and was lowest in AA women with TNBC (Figure **4**). Little is known about this transcript other than it is found on chromosome 2 in humans (2p11.2), is 3,653 bases long, shares 100% sequence with polymerase (RNA) I polypeptide A, 194kDa (POLR1A), and produces long, non-coding RNA (lncRNA) of undetermined function (NCBI Gene ID: 90784, updated on 16-Aug-2013). Non-coding RNA do not encode for protein, yet are alternatively spliced and processed into smaller products [59]. A search of miRBase (accessed March 13^th^, 2013) revealed that LOC90784 is not processed into any known microRNA [60]. However, lncRNAs are expressed in a disease-specific manner that makes them potential therapeutic targets since they are implicated in BRCa and promoting metastasis [61-63]. Another possibility is that LOC90784 is a miscategorized, alternatively spliced variant of POLR1A, but we have no evidence of this and further experiments are needed.

One emerging trend was that 26 genes and other transcripts were identified as differentially expressed in stage I, then 161 in stage II, and then 223 in stage III. This suggests that stage I tumors are more genetically similar to one another and that similarity is lost in higher stages. This increase in differentially expressed genes may be due to the increased rate of changes over time in a dysfunctional, cancerous cell due to genomic instability [64], but this finding may also suggest that AA women with BRCa have a higher rate of mutation than their CA counterparts. It would have been interesting to observe if this trend was maintained in stage IV tumors, but no stage IV AA data was available for analysis at the time of writing. 

This study further provides molecular insights on breast cancer health disparity and identifies several genes, other transcripts, and pathway targets for additional investigation. We observed increases in differentially expressed genes in later stages of cancer, suggesting higher genomic instability in African American patients. Several genes were differentially expressed including resistin, an adipocytokine involved in insulin resistance, obesity, and breast cancer, which had over four times higher expression in AA patients. A long, non-coding RNA, LOC90784, which may have protective activity in breast cancer, was significantly reduced in AA patients and was the lowest in TNBC. There was also higher expression in a number of signaling pathways related to cancer, such as aurora B, BRCA1, and p53, which suggests up- or down-stream dysregulation within these signal transduction pathways.

## Methods

### RNA-Seq Data from TCGA

Processed, de-identified, patient BRCa data (TCGA “Level 3” data designation) was downloaded from TCGA. The de-identified patient data included up to 20531 RNA sequence-derived gene expression values and clinical characteristics (e.g., age, cancer stage, receptor status). Custom Python scripts (The Python Software Foundation, http://www.python.org) were used to sort and aggregate patient data for analysis. Fifty-three AA tumor samples were matched by age and stage with 574 CA tumor samples, and a summary of the clinical characteristics of these patients is presented in Table **1**. The mean ages were 58 years for CA patients and 55 years for AA patients. Subtypes were assigned to patient samples according to receptor status from TCGA clinical data: luminal A (ER+ and/or PR+, HER2-), luminal B (ER+ and/or PR+, HER2+), HER2 (ER-, PR-, HER2+), and triple negative (ER-, PR-, HER2-). Normalized patient gene expression counts were used unless otherwise noted.

### Differential Gene Expression

DESeq version 1.10.1 was used to test for differentially expressed genes from patient data using the R statistical programming environment (version 2.15.2) [26,27]. DESeq uses the negative binomial distribution to derive a test for differential expression. The DESeq function “estimateDispersions()” was utilized with the argument sharingMode="gene-est-only" due to the relatively large number of patient samples, except for the HER2 comparison. Patient gene counts input into DESeq were rounded to the nearest whole number but not normalized. Jobs were run on the Florida State University High Performance Computing Cluster. 

### Pathway and Network Analysis

Differentially expressed genes identified by DESeq were input into PID (accessed February 11^th^, 2013) batch query tool and searched against the NCI-Nature curated data set [65]. PID identified pathways and constituent genes and assigned P-values to each pathway. The parameters used to generate P-values were the size of the query list, the number of molecules in a given pathway, and the number of molecules in the entire database. The P-value calculation for the batch query tool is based on a hypergeometric cumulative distribution function and was not corrected for multiple-hypothesis testing [66]. PID contains 137 pathways at the time of writing.

Gene Expression Network Analysis (GXNA) was used to identify subnetworks where differentially expressed genes are enriched compared to other parts of the whole gene/protein interaction network [67]. Gene expression data input into GXNA was first log_2_-transformed so that differences in means corresponded to fold-change, and default GXNA parameters were used. STRING, a database of protein-protein-interactions, was used to visualize GXNA results [68]. STRING takes a single gene product or list of gene products and returns a diagram of known and potential interactions. 

Gene Set Enrichment Analysis (GSEA) was used to identify differentially expressed gene sets including pathways [69,70]. Gene sets consist of groups of genes that may share common biological function, chromosomal location, or regulation. GSEA differs from network analysis in that it does not utilize topological or gene interaction information.

### False Discovery Rates (FDR)

A cutoff of P < 0.001 was used for reported results to reduce the number of false positives. If possible, the Benjamini-Hochberg procedure was used to control for FDR and is reported in the results [71]. Benjamini-Hochberg is used in DESeq output by default. GXNA adjusts for FDR using the more stringent family-wise error rate method. PID did not give a means to adjust for FDR, so Benjamini-Hochberg was again used to adjust the P-values. P-values that have been adjusted for FDR are referred to as adjusted P-values or P_adj_.

## Supporting Information

Table S1 **Log_2_ fold-change of differentially expressed genes and other transcripts across all comparisons.**
(XLSX)

Table S2 **Differentially expressed genes and other transcripts identified from the overall comparison using DESeq.**
(XLSX)

Table S3 A. Differentially expressed genes and other transcripts identified in stage I tumors with DESeq. B. Differentially expressed genes and other transcripts identified in stage II tumors with DESeq. C. Differentially expressed genes and other transcripts identified in stage III tumors with DESeq. D. Differentially expressed genes and other transcripts identified in luminal A tumors with DESeq. E. Differentially expressed genes and other transcripts identified in HER2 tumors with DESeq. F. Differentially expressed genes and other transcripts identified in triple negative tumors with DESeq.
(XLSX)
